# Malaria control in South America.

**DOI:** 10.3201/eid0502.990229

**Published:** 1999

**Authors:** P C Matteson


**Mary E. Wilson**
Mount Auburn Hospital, Cambridge, Massachusetts, USA; Harvard Medical School and Harvard School of Public Health, Boston, Massachusetts, USA

### References

1. Lederberg J, Shope RE, Oaks SC Jr., editors. Emerging infections: microbial threats to health in the United States. Institute of Medicine. Washington: National Academy Press, 1992.
2. Wilson ME, Levins R, Spielman A, editors. Disease in evolution: Global changes and emergence of infectious diseases. Vol 4. New York: Ann N Y Acad Sciences; 1994.

### Malaria Control in South America

**To the Editor:** The article by Roberts et al. regarding DDT use and malaria in South America (1) correctly observes that health policy makers have shifted the emphasis of malaria control programs from vector control to case detection and treatment and that malaria control has been woefully underfunded in recent years. However, their conclusions that increased malaria is due to decreased spraying of homes with DDT and that DDT is still needed for malaria control do not withstand close scrutiny.

The authors did not mention several factors influencing malaria increase in recent decades, including growing antimalarial-drug resistance, the deterioration of public health systems responsible for malaria control, and large-scale migration to areas at high risk for malaria (e.g., almost all Brazilian malaria cases occur in the Amazon region) (2,3). Extradomiciliary malaria transmission, poor housing conditions, and human behavior in frontier areas such as the Amazon region limit the usefulness of insecticides. Thus, the deduction of causality between less house spraying with DDT and increased malaria incidence under those circumstances is questionable.

Roberts et al. have not actually linked increased malaria with eliminating DDT use but rather with eliminating house spraying altogether, without implementing effective alternatives. Malaria's recent decline in Brazil is due to a strategy that combines health education, aggressive case detection and treatment, and environmental management to eliminate *Anopheles* breeding sites (C. Catão Prates, unpub. data). A similar strategy has sharply reduced malaria incidence and deaths in Colombia (W. Rojas, unpub. data). In Mexico, use of two synthetic pyrethroid insecticides (deltamethrin and lambda cyhalothrin) for bed-net treatment and house spraying is controlling malaria at a much lower cost than the use of the alternative insecticides tried earlier and mentioned by Roberts et al. (4). Far from being pursued "without meaningful debate," the reduction and phaseout of DDT and other persistent organic pollutants is the subject of a 3-year United Nations–facilitated global negotiation process begun in June 1998.

Roberts et al. assert that DDT applied indoors does not move easily from the application site; however, a mass balance model indicates that 60% to 80% of the DDT ends up outdoors within 6 months (K. Feltmate, A model and assessment of the fate and exposure of DDT following indoor application [bachelor's thesis]. Ontario: Trent University; 1998). From there, DDT can be transported long distances in air, waterborne sediments, and biota, accumulating in humans and other nontarget species (5). Meanwhile, residents of sprayed houses accumulate high, persistent body levels of DDT through skin contact and food contaminated with DDT from air and dust (6).

Long considered a probable human carcinogen, DDT also is associated with reduced lactation, premature births, absorbed fetuses, and lower birth weights (7-9). In addition, recent animal research has raised the possibility that exposure of human fetuses or infants to DDT may cause permanent behavioral changes and impairment of body systems (10-12).

Synthetic pyrethroid insecticides used on bed nets or for house spraying against malaria-infected mosquitoes seem safer for human health than DDT because humans and other mammals possess the ability to hydrolyze the pyrethroids rapidly and excrete them from the body (13-14). Nevertheless, DDT and pyrethroids share known health risks, notably endocrine disruption, and the possible transgenerational consequences of chronic human exposure to pyrethroids have not yet been studied (10,15-16). Optimal protection of human health requires the development of integrated malaria control strategies that eliminate or reduce routine insecticide use by taking maximum advantage of environmental management, biological controls, and other nonchemical vector control measures (17).

**Patricia C. Matteson**
U.N. Food and Agriculture Organization
Programme for Community Integrated Pest
Management in Asia, Hanoi, Vietnam

### References

1. Robert DR, Laughlin LL, Hshieh P, Legters LJ. DDT, global strategies, and a malaria control crisis in South America. Emerg Infect Dis 1997:3:295-302.
2. Wirth DF, Cattani J. Winning the war against malaria. Technology Review 1997;Aug/Sep:52-61.
3. World Resources Institute, United Nations Environment Programme, United Nations Development Programme, the World Bank. World Resources 1998-99. New York: Oxford University Press; 1998.
4. Matteson PC, editor. Disease vector management for public health and conservation. Washington: World Wildlife Fund-US; 1999.
5. Wania F, Mackay D. Tracking the distribution of persistent organic pollutants. Environmental Science & Technology News 1996;30:390-6.
6. Bouwman H, Cooppan RM, Becker PJ, Ngxongo S. Malaria control and levels of DDT in serum of two populations in Kwazulu. Journal of Toxicology and Environmental Health 1991;33:141-55.
7. Rogan WJ, Gladen BC, McKinney JD, Carreras N, Hardy P, Thullen J, et al. Polychlorinated biphenyls (PCBs) and dichlorodiphenyl dichloroethan (DDE) in human milk: effects on growth, morbidity, and duration of lactation. Am J Public Health 1987;177:1294-7.
8. Gladen BC, Rogan WJ. DDE and shortened duration of lactation in a northern Mexican town. Am J Public Health 1995;85:504-8.
9. Toxicological profile for 4,4'-DDE, 4,4'-DDD (updated). Atlanta: U.S. Department of Health and Human Services, Public Health Service, Agency for Toxic Substances and Diseases Registry; 1994. Pub. no. TP-93/05.
10. Eriksson P. Developmental neurotoxicity of environmental agents in the neonate. Neurotoxicology 1997;48:5719-26.
11. Rehana T, Rao PR. Effect of DDT on the immune system in Swiss albino mice during adult and perinatal exposure: humoral responses. Bull Environ Contam Toxicol 1992;48:535-40.
12. Banerjee BD, Saha S, Mohapatra TK, Ray A. Influence of dietary protein on DDT-induced immune responsiveness in rats. Indian J Exp Biol 1995;33:739-44.
13. Ray DE. Pesticides derived from plant and other organisms. In: Hayes WJ, Laws ER, editors. Handbook of pesticide toxicology. Vol 2. San Diego (CA): Academic Press; 1991. p. 585-636.
14. Casida JE, Gammon DW, Glickman AH, Lawrence LJ. Mechanisms of selective action of pyrethroid insecticides. Annu Rev Pharmacol Toxicol 1983;23:413-38.
15. Patro N, Misha SK, Chattopadhyay M, Patro IK. Neurological effects on deltamethrin on the postnatal development of cerebellum of rat. Journal of Biosciences 1997;22:117-30.
16. Smolen MJ, Sang S, Liroff RA. Hazards and exposures associated with DDT and synthetic pyrethroids used for vector control. Washington: World Wildlife Fund-US;1999.
17. Resolving the DDT dilemma: protecting biodiversity and human health. Toronto, Canada: World Wildlife Fund-Canada; 1998.

### Malaria Control in South America—Response to P.C. Matteson

**To the Editor:** Dr. Matteson, whose letter relies heavily on unpublished information and nonrefereed publications, states that growing drug resistance has contributed to increasing malaria. While drug resistance is important, when DDT use declined below effective levels (1), the proportion of *Plasmodium falciparum* infections (including infections with resistant strains) compared with *P. vivax* infections (no resistance) did not progressively increase (2). Moreover, malaria has increased in Central America, where drug resistance is unknown (3-6). As for attributing increasing malaria to deteriorating public health systems, the changes imposed on developing countries (in organizational structures of malaria control programs and prohibiting DDT [1,7]) correlate with increasing malaria rates (1).

Dr. Matteson states that large-scale migration explains why almost all Brazilian malaria cases occur in the Amazon Basin. However, DDT cleared malaria from the more populated and temperate southern regions of the country (8, unpublished report: U.S. Agency for International Development review in 1973-74 of Brazil's malaria eradication program). When DDT was in full use (pre-1980), large increases in malaria did not accompany population movement (1). With the 1970s' colonization program of the Basin came malaria problems, but not large population-based malaria increases. DDT prevented that (1,9-11). However, since DDT has been eliminated, persistent urban malaria is again becoming a problem (12-16).

Other factors (biting behavior, housing conditions, and human behavior), which Dr. Matteson attributes to increasing malaria, have always thwarted interdiction of malaria transmission in the Amazon Basin (17;18; an unpublished report: U.S. Agency for International Development review in 1973-74 of the malaria eradication program in Brazil) and are no more important today than they were before.

A UN-facilitated global negotiation process cited as a meaningful debate for malaria control is an effort to provide a legally binding agreement for global elimination of DDT and other persistent organic pollutants, not an open forum for debate of DDT use for malaria control.

